# Efeitos Agudos do Nitrato Dietético na Pressão Central e Desempenho Cardíaco em Hipertensos: Estudo Cruzado, Randomizado e Placebo-Controlado

**DOI:** 10.36660/abc.20220209

**Published:** 2022-12-20

**Authors:** Samanta Mattos, Michelle Rabello Cunha, Bianca Cristina Marques, Jenifer d´El-Rei, Diego dos Santos Baião, Vania M. F. Paschoalin, Wille Oigman, Mario Fritsch Neves, Fernanda Medeiros

**Affiliations:** 1 Departamento de Clínica Médica Universidade do Estado do Rio de Janeiro Rio de Janeiro RJ Brasil Departamento de Clínica Médica – Universidade do Estado do Rio de Janeiro (UERJ), Rio de Janeiro, RJ – Brasil; 2 Instituto de Química Universidade Federal do Rio de Janeiro Rio de Janeiro RJ Brasil Instituto de Química – Universidade Federal do Rio de Janeiro (UFRJ), Rio de Janeiro, RJ – Brasil; 3 Escola de Nutrição Universidade Federal do Estado do Rio de Janeiro Rio de Janeiro RJ Brasil Escola de Nutrição da Universidade Federal do Estado do Rio de Janeiro (UNIRIO), Rio de Janeiro, RJ – Brasil

**Keywords:** Beta Vulgaris, Hipertensão, Endotélio, Óxido Nítrico

## Abstract

**Fundamento:**

O nitrato inorgânico (NO_3_^–^) da dieta pode fornecer substrato fisiológico para reduzir o nitrito (NO_2_^–^) a óxido nítrico (NO) independente do endotélio. Estudos sugerem que o NO_3_^–^ inorgânico tem efeitos benéficos na saúde cardiovascular.

**Objetivos:**

Este estudo avaliou os efeitos agudos de 500 mL de suco de beterraba rico em nitrato (SB; contendo 11,5mmol NO_3_^–^) na pressão arterial e na função endotelial em pacientes hipertensos tratados.

**Métodos:**

Estudo cruzado, randomizado, controlado por placebo foi realizado em pacientes hipertensos tratados (n=37; mulheres=62%) que foram submetidos à avaliação clínica e nutricional, avaliação dos parâmetros hemodinâmicos centrais e reatividade microvascular. O nível de significância foi p<0,05.

**Resultados:**

A média de idade foi 59±7 anos e das pressões sistólica e diastólica foi de 142±10/83±9 mmHg. Houve aumento significativo na taxa de viabilidade subendocárdica (RVSE; 149±25 vs. 165±30%, p<0,001) e redução na duração da ejeção (DE; 37±4 vs. 34±4%, p<0,001) na fase beterraba, mas nenhuma diferença significativa de RVSE na fase controle. O % de aumento na perfusão (155 vs. 159%, p=0,042) cresceu significativamente na fase beterraba, o que não foi observado na fase controle. Na fase beterraba, a alteração da RVSE apresentou correlação significativa com a alteração da área sob a curva de hiperemia reativa pós-oclusiva (ASC-HRPO) (r=0,45, p=0,012). A mudança na DE mostrou uma correlação significativa com pico de perfusão pós-intervenção (r=-0,37, p=0,031) e ASC-HRPO (r=-0,36, p=0,046).

**Conclusão:**

A ingestão aguda de SB por pacientes hipertensos resultou em melhora da função endotelial, que foi associada à maior viabilidade subendocárdica e desempenho na contração miocárdica.

## Introdução

A doença cardiovascular (DCV) é a principal causa de morte em todo o mundo. Cerca de 17,9 milhões de pessoas morreram em 2019, representando 32% de todas as mortes.^1^ O endotélio é um dos principais reguladores da homeostase vascular, desempenha papel na modulação do tono vascular sintetizando e liberando fatores de relaxamento derivado do endotélio, incluindo óxido nítrico (NO).^2^O desequilíbrio dessas substâncias leva à disfunção endotelial,^3^ que é marcador de remodelação vascular e função vascular prejudicada.^4^

A função microvascular coronariana é um indicador de oferta e demanda de oxigênio miocárdico, avaliado pelo índice de viabilidade subendocárdica (RVSE), apresentando uma estimativa da perfusão miocárdica em relação à carga de trabalho cardíaca e um preditor da reserva de fluxo coronariano.^5 , 6^ Valores de RVSE baixo em pacientes hipertensos foi associado com reserva de fluxo coronariano reduzida.^5^

Os hábitos alimentares influenciam vários mecanismos envolvidos com fatores de risco cardiovascular.^7^ O teor de nitrato inorgânico (NO_3_‾) em vegetais de raiz pode fornecer um substrato fisiológico para redução a nitrito (NO_2_‾), NO e outros produtos metabólicos via NO_3_-NO_2_-NO.^8^ Dentre as moléculas mais importantes produzidas no sistema cardiovascular que mantêm a homeostase vascular, a biodisponibilidade do NO tem grande relevância na patogênese das DCV.^9^

Os vegetais são os principais contribuintes dietéticos de NO_3_‾,^10 , 11^e a beterraba (Beta vulgaris) é rica em NO_3_‾ inorgânico.^12^ A beterraba tem sido destacada como um suplemento multidirecionado na disfunção vascular, aterosclerose e diabetes, e tem sido considerada no tratamento complementar da hipertensão.^13^

Muitos estudos têm demonstrado o efeito benéfico do suco de beterraba (SB) na pressão arterial (PA), bem como melhora da função endotelial e redução da rigidez arterial.^14 , 15^ No entanto, até o momento, não há estudos avaliando o efeito agudo de SB na PA periférica e central, parâmetros hemodinâmicos e reatividade microvascular ao mesmo tempo. Este estudo teve como objetivo avaliar os efeitos agudos da ingestão dietética de NO_3_‾ sobre a PA e a função endotelial em pacientes hipertensos tratados.

## Métodos

### Participantes

Pacientes hipertensos com idade entre 40 e 70 anos, de ambos os sexos, em uso regular de anti-hipertensivos, foram selecionados em nosso ambulatório (RJ) e admitidos em um estudo randomizado, cruzado, placebo-controlado. Os critérios de exclusão foram hipertensão secundária; uso de betabloqueadores ou estatinas, diabetes mellitus ou terapia de reposição hormonal. O protocolo foi aprovado pelo Comitê de Ética local e todos os participantes leram e assinaram o termo de consentimento livre e esclarecido. Este ensaio foi registrado no ClinicalTrials.Gov (NCT04020796).

### Desenho do estudo

**Figura 1:**
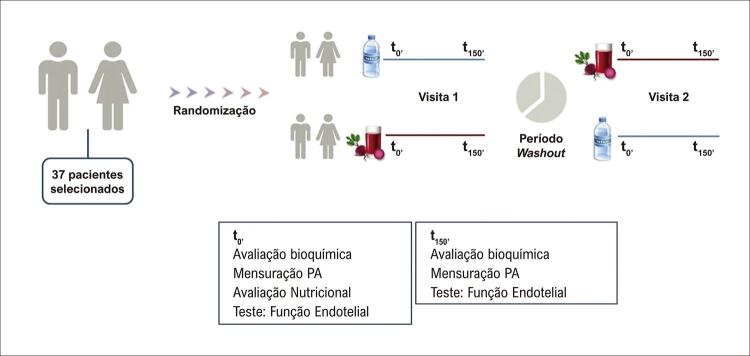
Fluxograma de projeto de estudo. PA: pressão arterial.

Na primeira consulta (basal/t0’), os pacientes foram submetidos à avaliação da PA, antropometria e exames. O site *randomization.com* foi utilizado para gerar a ordem de randomização das intervenções, o que foi feito por um pesquisador que não participou diretamente dos procedimentos do estudo (randomização cega).^16^Cada participante foi randomizado para as intervenções cruzadas. Os pacientes receberam sua respectiva intervenção, SB ou água, e permaneceram em repouso por 150 minutos, tempo de pico de NO_3_‾ e NO_2_‾ na circulação sanguínea.^17^Ao final desse período (t150’), os exames foram novamente realizados. Após o período de *washout* de 7 dias, os pacientes foram submetidos à intervenção alternada ( Figura 1 ).

### Intervenção

As beterrabas foram adquiridas em supermercado local (localizado no município do Estado do Rio de Janeiro, Brasil), e foram pesadas, higienizadas, descascadas, fracionadas e liquefeitas por centrifugação de alimentos sem adição de água. O volume final de SB foi de 500 mL. A água (Minalba®, Brasil), bebida controle, continha < 0,001 mmol NO_3_‾ em 500 mL. A bebida controle foi escolhida com base em alguns estudos que utilizaram a água como controle da intervenção devido ao seu baixo teor de nitrato.^17^O NO_3_‾ e NO_2_‾ do SB foram quantificados e suas dosagens séricas usadas como marcadores indiretos de produção de NO foram avaliados conforme previamente descrito.^18 , 19^

### Avaliação bioquímica

Amostras de sangue venoso foram coletadas após jejum de 8 horas, antes de qualquer intervenção. Glicose sérica, colesterol total, colesterol da lipoproteína de alta densidade (HDL) e triglicerídeos (TG) foram medidos usando uma técnica de AutoAnalyzer (Technicon DAX96, Miles Inc). As concentrações de colesterol da lipoproteína de baixa densidade (LDL) foram calculadas usando a equação de Friedewald, quando as concentrações de TG <400 mg/dl.^20^ A avaliação da função renal foi realizada por meio da estimativa da taxa de filtração glomerular, pela equação Chronic Kidney Disease - Epidemiology Collaboration (CKD-EPI).^21^

### Avaliação antropométrica

Os parâmetros antropométricos foram avaliados por meio de medidas de peso corporal (kg) e estatura (metros) em balança eletrônica com estadiômetro (Filizola SA, São Paulo, SP, Brasil), e o índice de massa corporal (IMC) foi calculado e expresso em kg/m^2^.

### Pressão arterial e avaliação de risco cardiovascular

As medidas de PA sistólica (PAS) e PA diastólica (PAD) foram obtidas usando um dispositivo eletrônico calibrado (modelo HEM-705CP, OMRON Healthcare Inc., Illinois). Após três leituras com intervalo de um minuto, a média foi calculada e considerada para análise do estudo. A estimativa de risco cardiovascular e idade vascular foram baseadas no Framingham Heart Study.^22^

### Reatividade microvascular

A reatividade microvascular foi avaliada usando um sistema Laser Speckle Contrast Image (Pericam PSI System, Perimed, Suécia) em combinação com hiperemia reativa pós-oclusiva (HRPO) para redução contínua de alterações de perfusão cutânea dependentes do endotélio microvascular expressas em unidades arbitrárias de perfusão (UAP). Um esfigmomanômetro foi usado na artéria braquial para aplicar uma pressão de 50 mmHg acima da PAS por três minutos. Após descompressão rápida, as mudanças de fluxo foram registradas para avaliar HRPO. Perfusão aumentada (%): (pico - perfusão basal) /perfusão basal x 100. Aumento da área sob a curva (ASC) (%): (HRPO -ASC - ASC basal) /ASC basal x 100.

### Parâmetros hemodinâmicos centrais

A avaliação da reflexão da onda arterial foi realizada de forma não invasiva com um aparelho de tonometria (SphygmoCor, AtCor Medical, Sydney, Austrália). O sistema SphygmoCor usa uma função de transferência generalizada validada para gerar as pressões aórticas centrais correspondentes após a aquisição de 10 formas de onda sequenciais. Pressão sistólica aórtica (aoPS), pressão de pulso aórtico (aoPP), pressão de aumento (AP), índice de aumento (AIx), RVSE e duração de ejeção (DE) derivadas da análise da forma de onda do pulso. A RVSE é um índice de viabilidade subendocárdica que já foi comparada com métodos invasivos e considerada uma medida de perfusão miocárdica relativa à sobrecarga cardíaca. Na análise da onda de pulso, a RVSE é definida como: RVSE = área aórtica diastólica/ área aórtica sistólica.^6 , 23^

### Análise estatística

Para determinação do tamanho amostral deste estudo, foi considerada a equivalência da variação da dilatação mediada pelo fluxo (DMF), observada no estudo de Bakker e cols (2015).^24^Desse modo, para uma diferença de 1,4% na DMF, desvio-padrão de 1,9, 80% poder do estudo e significância em 5%, seria necessário um número mínimo de 30 participantes. Os resultados foram expressos como média ± desvio padrão (DP) para variáveis contínuas com distribuição normal, ou mediana (intervalo interquartil) para variáveis contínuas não gaussianas. O teste de Shapiro-Wilk foi utilizado para avaliar a distribuição normal. Os grupos, controle e intervenção foram comparados pelo teste t pareado em variáveis com distribuição normal e realizado o teste de Wilcoxon para variáveis com distribuição não-normal. As variáveis categóricas foram apresentadas como frequência e porcentagem. O coeficiente de Pearson foi obtido nos testes de correlação entre as variáveis contínuas. O nível de significância adotado na análise estatística foi de 5%. A análise estatística foi realizada por meio do Statistical Package for the Social Sciences (SPSS)® versão 20 para Windows (SPSS, Chicago, IL).

## Resultados

Para este estudo, foram selecionadas 37 pacientes com idade média de 59±7 anos foram incluídos no estudo. Após a avaliação antropométrica e clínica, observou-se que a maioria estava acima do peso com IMC 29±4 kg/m^2^, sexo feminino (62%), apresentando risco cardiovascular intermediário (14%), PAS>140 mmHg, PAD>80 mmHg, e colesterol total elevado ( Tabela 1 ). As classes de anti-hipertensivos mais utilizadas foram os inibidores do sistema renina-angiotensina (48%), diurético tiazídico (36%) e bloqueador dos canais de cálcio (BCC; 16%).

**Tabela 1:** Características basais dos sujeitos do estudo

																					Amostra total (n= 37)
Idade (anos)																					59 ± 7
Risco cardiovascular (%)																					14 (10 - 22)
Idade vascular (anos)																					76 (67 - 86)
Gênero ♂ (%)																					38
♀ (%)																					
Índice de massa corporal (Kg/m^2^)																					29 ± 4
Circunferência cintura (cm) ♂																					98 ± 8
♀																					92 ± 11
PA sistólica (mmHg)																					142 ± 10
PA diastólica (mmHg)																					83 ± 9
Pressão pulso (mmHg)																					59 ± 11
Pressão arterial média (mmHg)																					103 ± 9
**Variáveis bioquímica**																					
Total colesterol (mg/dl)																					203 ± 38
HDL-colesterol (mg/dl)																					56 ± 20
LDL-colesterol (mg/dl)																					121 ± 30
Triglicerídeos (mg/dl)																					110 (78 - 178)
Glicose (mg/dl)																					90 ± 8
CKD-EPI (ml/min/1,73m^2^)																					80 (67 - 98)
**Tratamento anti-hipertensivo, n (%)**																					
Bloqueador do receptor de angiotensina																					29 (78)
Inibidor da enzima conversora de angiotensina																					7 (19)
Diurético tiazídico																					27 (73)
Bloqueador do canal de cálcio																					12 (32)
Monoterapia																					8 (22)
2 drogas																					20 (54)
3 drogas																					9 (24)

*Resultados expressos em média (±desvio padrão), mediana (intervalo interquartil) ou proporção nas variáveis categóricas. PA: pressão arterial; HDL: lipoproteína de alta densidade; LDL: lipoproteína de baixa densidade; CKD-EPI: Chronic Kidney Disease – Epidemiology Collaboration.*

O SB apresentou altos teores de NO_3_‾ e NO_2_‾ em sua composição. A análise sérica dos teores de NO_3_‾ e NO_2_‾ nas fases controle e beterraba antes e após cada intervenção está descrita na Tabela 2 . Não foram observadas diferenças significativas nas concentrações séricas de NO_3_‾ e NO_2_‾ antes da ingestão de SB e água. No entanto, houve aumento significativo nos níveis séricos de NO_3_‾ e NO_2_‾ após intervenção com SB. Este aumento foi aproximadamente três vezes o valor basal desta fase.

**Tabela 2:** Nitrato e nitrito séricos – Fase de controle e beterraba

	Controle		Valor de p	Beterraba		Valor de p
	
	Basal (n=24)	Pós (n=24)		Basal (n=28)	Pós (n=28)	
Nitrato (µmol/L)	56,4 ± 26,5	65,3 ± 34,9	0,063	59,7 ± 13,6	169,4 ± 76,9	< 0,001
Nitrito (µmol/L)	0,100 ± 0,012	0,095 ± 0,018	0,162	0,099 ± 0,014	0,336 ± 0,159	< 0,001

*Dados expressos em média±desvio padrão. Valor de p correspondente ao teste t pareado.*

Um aumento significativo da PAS periférica foi observado na fase controle, mas não na fase beterraba. Houve também aumento da aoPS na fase controle e em menor grau na fase beterraba. A fase beterraba apresentou redução significativa na DE e aumento na RVSE ( Tabela 3 ).

**Tabela 3:** Medidas de pressão arterial periférica e parâmetros hemodinâmicos centrais – Fase de controle e beterraba

	Controle		Valor de p	Beterraba		Valor de p
	
	Basal (n=37)	Pós (n=37)		Basal (n=37)	Pós (n=37)	
**Pressão arterial periférica**						
PA Sistólica (mmHg)	139 ± 9	144 ± 15	0,044	138 ± 13	139 ± 17	0,621
PA Diastólica (mmHg)	83 ± 9	84 ± 9	0,268	84 ± 11	85 ± 11	0,492
Pressão Pulso (mmHg)	56 ± 10	60 ± 13	0,039	54 ± 10	55 11	0,905
PAM (mmHg)	102 ± 8	104 ± 9	0,093	102 ± 11	103 ± 12	0,532
**Parâmetros hemodinâmicos centrais por tonometria de aplanação**						
aoSP (mmHg)	137 ± 15	143 ± 14	0,003	132 ± 15	136 ± 16	0,061
aoPP (mmHg)	52 ± 12	56 ± 13	0,007	56 ± 26	57 ± 24	0,736
AP (mmHg)	19 ± 7	21 ± 9	0,009	17 ± 9	19 ± 8	0,278
Augmentation Index (%)	36 (32 - 40)	38 (32 - 43)	0,070	35 (28 - 39)	37 (31 - 41)	0,082
AIx@75 (%)	30 (27 - 34)	32 (26 - 37)	0,442	29 (24 - 34)	31 (24 - 34)	0,751
Duração de ejeção (%)	35 ± 4	34 ± 4	0,019	37 ± 4	34 4	<0,001
RVSE (%)	155 ± 28	160 ± 28	0,080	149 ± 25	165 ± 30	<0,001

*Dados expressos em média±desvio padrão ou mediana (intervalo interquartil) quando apropriado. Valor de p na comparação entre os valores basais e pós-intervenção, correspondente ao teste t pareado para as variáveis com distribuição normal e teste de Wilcoxon para variáveis com distribuição não-normal. PA: pressão arterial; PAM: pressão arterial média; aoPS: pressão sistólica aórtica; aoPP: pressão de pulso aórtica; AP: pressão de aumento; Alx@75: Índice de Aumento corrigido para frequência cardíaca de 75 batimentos por minuto; RVSE: razão de viabilidade subendocárdica.*

No teste HRPO, a fase beterraba demonstrou aumento significativo no % de perfusão. A porcentagem de aumento da ASC de perfusão cutânea induzida pela HRPO na fase controle diminuiu após a ingestão de água (bebida controle) e aumentou após a ingestão de SB mas não alcançando significância estatística ( Tabela 4 ).

**Tabela 4:** Medidas de avaliação da função endotelial por reatividade microvascular – Controle e fase beterraba

	Controle		Valor de p	Beterraba		Valor de p
	
	Basal (n=37)	Pós (n=37)		Basal (n=37)	Pós (n=37)	
Perfusão basal (UAP)	30 ± 12	27 ± 10	0,097	33 ± 11	29 ± 9	0,005
Pico (UAP)	83 ± 22	73 ± 23	0,001	85 ± 24	76 ± 21	0,005
Perfusão de aumento (%)	177 (132 - 243)	148 (102 - 212)	0,722	155 (125 - 190)	159 (121 - 227)	0,042
Aumento ASC (%)	65 ± 34	63 ± 36	0,816	67 ± 28	73 ± 25	0,182

*Dados expressos em média±desvio padrão ou mediana (intervalo interquartil) quando apropriado. Valor de p na comparação entre os valores basais e pós-intervenção, correspondente ao teste t pareado para as variáveis com distribuição normal e teste de Wilcoxon para variáveis com distribuição não-normal. UAP: unidade arbitrária de perfusão; ASC: área sob a curva.*

**Figura 2:**
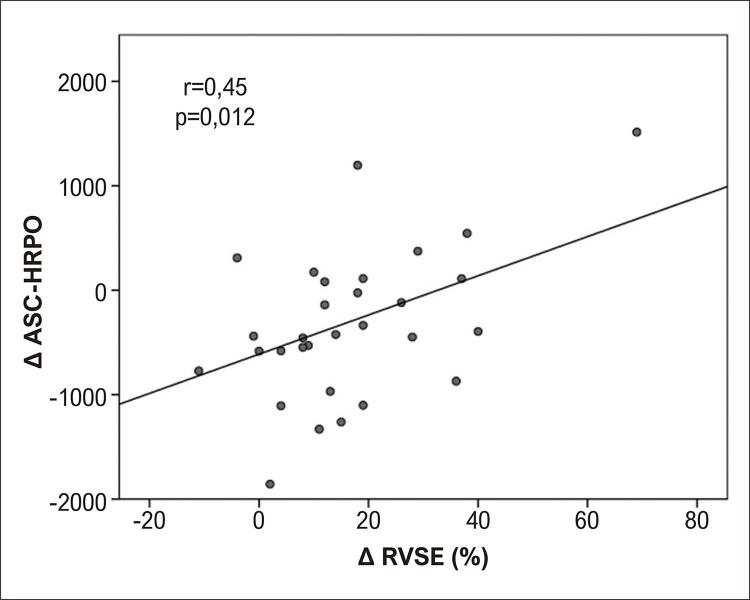
Correlação no grupo beterraba entre alteração na razão de viabilidade subendocárdica (∆ RVSE) e alteração na área sob a curva de hiperemia reativa pós-oclusiva (∆ ASC-HRPO).

**Figura 3:**
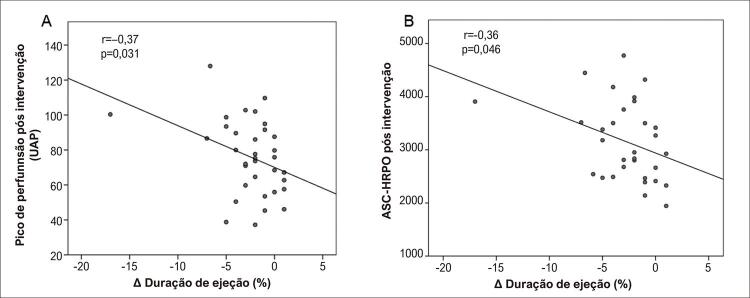
Correlação no grupo beterraba de mudança (∆) na duração da ejeção com o pico pós-intervenção (A) e com a área pós-intervenção sob a curva de hiperemia reativa pós-oclusiva (ASC-HRPO) (B).

Na fase beterraba, a mudança no RVSE correlacionou-se positivamente com a mudança na AUC-HRPO ( Figura 2 ). A DE na fase beterraba observou correlações inversas com parâmetros de função endotelial, pico pós-intervenção (A) e ASC-HRPO (B ( Figura 3 ).

## Discussão

O presente estudo foi realizado para determinar os efeitos agudos da ingestão dietética de NO_3_‾ através do SB, rico em NO_3_‾ inorgânico, na PA e na função endotelial em pacientes hipertensos tratados. Entre os principais resultados, observou-se atenuação dos níveis pressóricos periféricos e centrais, redução da DE, aumento da RVSE e melhora da função vascular, associados a elevação sérica de NO_3_‾ e NO_2_‾ após ingestão única de NO_3_‾ inorgânico.

Neste estudo, o SB utilizado na fase de intervenção apresentou altos níveis de NO_3_‾ e NO_2_‾ em sua composição. Usamos vegetais frescos para preparar o SB em vez de comprar um suco comercial mais caro. Vários estudos utilizaram SB industrializado com concentração de NO_3_‾ semelhante, mas teor de NO_2_‾ inferior ao observado no presente estudo.^17 , 25^ Além disso, a concentração de NO_3_‾ foi quase 1,5 vezes maior do que no suco não industrializado utilizado em outro estudo recente.^26^

Os níveis séricos de NO_3_‾ e NO_2_‾ aumentaram significativamente após 150 minutos de intervenção com SB neste estudo. Webb et al.^17^ avaliaram a ingestão única de 500 mL de SB e encontraram um rápido aumento (16 vezes) na concentração de NO_3_‾ circulante após os primeiros 30 minutos, com pico de 1,5 hora e permanecendo nesse nível até 6 horas após a ingestão. A proporção de aumento de NO_3_‾ circulante foi semelhante em comparação com o teor de NO_3_‾ deste estudo.

Houve aumento significativo da PA periférica na fase controle, que foi atenuada na fase de intervenção após a ingestão de SB. A maioria dos estudos em indivíduos normotensos avaliou o consumo crônico de SB e apresentou diminuição dos níveis de PA.^27 , 28^Poucos estudos avaliaram os efeitos do consumo de SB por hipertensos.^15 , 29^ Kapil et al.^30^ avaliaram a ingestão de SB por quatro semanas em hipertensos com e sem tratamento anti-hipertensivo e encontraram redução nos valores de PAS e PAD em relação aos valores basais. Kerley et al.^31^ relataram reduções significativas nos níveis pressóricos avaliados pela monitorização ambulatorial da pressão arterial (MAPA) de 24 horas após ingestão crônica de SB em hipertensos tratados. No entanto, Bondonno et al. (2015) não observaram alterações na PA e MAPA de consultório ao avaliar hipertensos tratados com ingestão de SB por uma semana.^29^

Esses resultados controversos em hipertensos podem ser atribuídos à grande heterogeneidade do desenho dos estudos em relação ao volume de SB (140mL a 250mL), teor de NO_3_ **‾** (6,8 a 12,9 mmol), tempo de suplementação e tratamento anti-hipertensivo. Alguns agentes farmacológicos, como inibidores do sistema renina-angiotensina e BCC, podem causar vasodilatação por influenciar a síntese de NO,^32^ o que poderia atenuar os efeitos do SB sobre os níveis de PA nos pacientes tratados neste estudo. Outro fator a ser considerado é apresentado em uma metanálise recente, na qual os autores demonstram que os efeitos hipotensores da beterraba podem ser afetados por doenças crônicas. Uma maior redução na PAS e PAD foi observada após a suplementação de beterraba em participantes não saudáveis do que em participantes saudáveis. Além disso, indivíduos com sobrepeso e obesos tiveram uma resposta mais alta semelhante à suplementação de SB.^33^

O comportamento da PA central foi semelhante ao da PA periférica com aumento significativo na fase controle, o que não foi observado na fase beterraba. Até o momento, não há estudos avaliando a PA central após a ingestão de NO_3_‾ inorgânico em indivíduos com hipertensão. De fato, alguns estudos que avaliaram a PA central após ingestão aguda de SB foram realizados em indivíduos normotensos, mostrando uma redução significativa da PA aórtica.^34 , 35^

Neste estudo, a DE apresentou redução significativa após a ingestão de SB. Hughes et al.^34^ avaliaram os efeitos agudos da ingestão de SB por mulheres normotensas e encontraram redução gradual da DE após duas horas da ingestão. A redução na DE está relacionada a uma aorta menos rígida e uma diminuição na pós-carga cardíaca, e a ingestão de SB pareceu melhorar a complacência vascular, o que pode facilitar o desempenho cardíaco.^36^

RVSE é um marcador sensível de oferta e demanda subendocárdica de oxigênio que se correlaciona com isquemia miocárdica.^37^ Quanto menor a razão de viabilidade, menor a perfusão cardíaca, o que pode estar relacionado à rigidez arterial. Os mediadores inflamatórios participam ativamente nos mecanismos de lesão vascular e estão aumentados em todos os estágios da hipertensão, e essa associação acelera o processo de envelhecimento vascular.^38^Neste estudo, o RVSE aumentou significativamente após a ingestão de SB, e nenhuma alteração foi observada na fase controle. Até onde sabemos, não existem estudos abordando RVSE em hipertensos submetidos à ingestão de SB. De acordo com este resultado, Hughes et al.^34^ avaliaram os efeitos agudos da ingestão de SB por mulheres jovens e normotensas na pós-menopausa e observaram um aumento significativo na RVSE após 150 e 180 minutos de ingestão de bebida.

A reatividade microvascular medida pelo percentual de aumento da perfusão no HRPO mostrou um aumento significativo após a ingestão de SB, demonstrando uma melhora na função endotelial. Até o momento, não existem protocolos clínicos avaliando os efeitos do NO_3_‾ dietético na reatividade microvascular utilizando esta metodologia em indivíduos hipertensos. Em um estudo realizado em indivíduos com excesso de peso, três semanas de suplementação de 70ml de SB não apresentaram diferença significativa no pico e no índice HRPO.^39^

Algumas limitações deste estudo devem ser consideradas. Os critérios de exclusão dificultaram a inscrição dos participantes, principalmente quanto ao uso de betabloqueadores e estatinas, medicamentos amplamente utilizados em hipertensos devido às demais comorbidades. No entanto, a população do estudo foi mais numerosa do que o tamanho estimado da amostra e semelhante aos demais estudos. O período de intervenção de 150 minutos no período de jejum pode ter influenciado no aumento da PA, que foi mais evidente na fase controle. Por outro lado, esse efeito contribuiu para uma melhor observação da ação do NO_3_‾ da dieta sobre os níveis de PA. Por fim, realizamos uma intervenção de dose única e, portanto, nossos achados não podem ser comparados com efeitos de médio e longo prazo.

## Conclusão

A ingestão de SB resultou em benefícios agudos sobre os parâmetros vasculares em indivíduos hipertensos, levando a uma maior viabilidade subendocárdica, maior desempenho na contração miocárdica e melhora da função endotelial. Este foi o primeiro estudo que aplicou diferentes métodos para avaliar parâmetros vasculares e demonstrou efeitos benéficos da ingestão única de SB em adultos hipertensos tratados. No entanto, mais estudos são necessários para avaliar a eficácia da via NO_3_-NO_2_-NO, especialmente em indivíduos com hipertensão e outros fatores de risco para doenças cardiovasculares.
