# Supplementary material for: Efeitos Agudos do Nitrato Dietético na Pressão Central e Desempenho Cardíaco em Hipertensos: Estudo Cruzado, Randomizado e Placebo-Controlado
Source: Arq Bras Cardiol. 2022 Dec 20;120(1):e20220209. [Article in Portuguese] doi: 10.36660/abc.20220209 (PMC9833313; doi:10.36660/abc.20220209)
Supplement: Translation

# Translation: Acute Effects of Dietary Nitrate on Central Pressure and Endothelial Function in Hypertensive Patients: A Randomized, Placebo-Controlled Crossover Study

**Authors:** Samanta Mattos, Michelle Rabello Cunha, Bianca Cristina Marques, Jenifer d´El-Rei, Diego dos Santos Baião, Vania M. F. Paschoalin, Wille Oigman, Mario Fritsch Neves, Fernanda Medeiros

**Affiliations:** 1 Brazil Departamento de Clínica Médica – Universidade do Estado do Rio de Janeiro (UERJ), Rio de Janeiro, RJ – Brazil; 2 Brazil Instituto de Química – Universidade Federal do Rio de Janeiro (UFRJ), Rio de Janeiro, RJ – Brazil; 3 Brazil Escola de Nutrição da Universidade Federal do Estado do Rio de Janeiro (UNIRIO), Rio de Janeiro, RJ – Brazil

**Keywords:** Beta Vulgaris, Hypertension, Endothelium, Nitric Oxide

## Abstract

**Background:**

The diet’s inorganic nitrate (NO_3_^–^) may provide a physiological substrate for reducing nitrate (NO_2_^–^) to NO independent of the endothelium. Studies suggest that inorganic NO_3_^–^has beneficial effects on cardiovascular health.

**Objective:**

This study evaluated the acute effects of 500 mL nitrate-rich beetroot juice (BRJ; containing 11.5mmol NO_3_^–^) on blood pressure and endothelial function in treated hypertensive patients.

**Methods:**

A randomized, placebo-controlled, crossover study was conducted in treated hypertensive patients (n=37; women=62%) who underwent clinical and nutritional evaluation and assessment of central hemodynamic parameters and microvascular reactivity. The significance level was p<0.05.

**Results:**

The mean age was 59±7 years, and mean systolic and diastolic blood pressures were 142±10/83±9mmHg. There was a significant increase in the subendocardial viability ratio (SEVR; 149±25 vs. 165±30%, p<0.001) and reduction in ejection duration (ED; 37±4 vs. 34±4%, p<0.001) in the beetroot phase but no significant SEVR difference in the control phase. The % increase in perfusion (155 vs. 159 %, p=0.042) was significantly increased in the beetroot phase, which was not observed in the control phase. In the beetroot phase, the change in SEVR showed a significant correlation with the change in the area under the curve of post-occlusive reactive hyperemia (AUC-PORH) (r=0.45, p=0.012). The change in ED showed a significant correlation with the post-intervention perfusion peak (r=-0.37, p=0.031) and AUC-PORH (r=-0.36, p=0.046).

**Conclusions:**

The acute ingestion of BRJ by hypertensive patients resulted in an improvement of endothelial function, which was associated with higher subendocardial viability and performance in myocardial contraction.

## Introduction

Cardiovascular disease (CVD) is the leading cause of death worldwide, with around 17.9 million people dying in 2019, accounting for 32% of all deaths.^1^The endothelium is one of the main regulators of vascular homeostasis; it plays a role in modulating vascular tone by synthesizing and releasing endothelium-derived relaxation factors, including nitric oxide (NO).^2^The imbalance of these substances leads to endothelial dysfunction,^3^which is a marker of vascular remodeling and impaired vascular function.^4^

The coronary microvascular function is an indicator of myocardial oxygen supply and demand assessed by the subendocardial viability ratio (SEVR), presenting an estimate of myocardial perfusion concerning cardiac workload and a predictor of coronary flow reserve.^5 , 6^Low SEVR values in hypertensive patients were associated with reduced coronary flow reserve.^5^

Eating habits influence several mechanisms involved with cardiovascular risk factors.^7^The inorganic nitrate (NO_3_‾) content in root vegetables can provide a physiological substrate for the reduction to nitrite (NO_2_‾), NO, and other metabolic products via the NO_3_-NO_2_-NO.^8^Among the most important molecules produced in the cardiovascular system that maintains vascular homeostasis, NO bioavailability has great relevance in the pathogenesis of CVD.^9^

Vegetables are the main dietary contributors of NO_3_‾,^10 , 11^and beetroot ( *Beta vulgaris* ) is rich in inorganic NO_3_‾.^12^Beetroot has been highlighted as a multitargeted supplement in vascular dysfunction, atherosclerosis, and diabetes and has been considered a complementary treatment for hypertension.^13^

Many studies have shown the beneficial effect of beetroot juice (BRJ) on blood pressure (BP), improving endothelial function and reducing arterial stiffness.^14 , 15^However, to date, no studies have evaluated the acute effect of BRJ on BP peripheral and central, hemodynamic parameters and microvascular reactivity simultaneously. This study aimed to evaluate the acute effects of dietary NO_3_‾ intake on BP and endothelial function in treated hypertensive patients.

## Methods

### Participants

Hypertensive patients aged between 40 and 70 years, of both genders, in regular use of antihypertensive drugs, were selected from our outpatient clinic (Rio de Janeiro city, Brazil) and admitted to a randomized, crossover, placebo-controlled study. The exclusion criteria were secondary hypertension, use of beta-blockers or statin, diabetes mellitus or hormone replacement therapy. The local Ethics Committee approved all procedures, and all the participants signed the informed consent. This trial was registered at ClinicalTrials.Gov (NCT04020796).

### Study design

On the first visit (baseline/t0’), patients underwent evaluation of BP, anthropometric, laboratory testing, and tests. The website *randomization.com* was used to generate the randomization order of the interventions, which was done by a researcher who did not directly participate in the study procedures (blind randomization).^16^Each participant was randomized to the crossover interventions. Patients were then provided their respective intervention, BRJ or water, and remained at rest for 150 min, which is the peak time of NO_3_‾ and NO_2_‾ in blood circulation.^17^At the end of this period (t150’), the exams were performed again. After the 7-day washout period, the patients underwent alternating interventions ( Figure 1 ).

**Figure 1:** Study design flowchart. BP: blood pressure.

### Intervention

The beetroots were purchased from a local supermarket (located in the municipality of Rio de Janeiro State, Brazil) and were weighed, sanitized, peeled, fractionated and liquefied by a food centrifuge without the addition of water. The final volume of BRJ offered to each patient was 500 mL. Water (Minalba®, Brazil), used as a control drink, contained < 0.001 mmol NO_3_‾ in 500 mL. The control drink was chosen based on some studies that used water as a control intervention due to its low nitrate content.^17^The NO_3_‾ and NO_2_‾ of BRJ were quantified, and their serum levels used as indirect markers of NO production were evaluated as previously described.^18 , 19^

### Biochemical evaluation

Venous blood samples were collected after 8-hour fasting before any intervention. Serum glucose, total cholesterol, high-density lipoprotein (HDL)-cholesterol and triglycerides (TG) were measured using an AutoAnalyzer technique (Technicon DAX96, Miles Inc). Low-density lipoprotein (LDL)-cholesterol concentrations were calculated using Friedewald’s equation when TG concentrations <400 mg/dl.^20^The evaluation of renal function was performed using the estimated glomerular filtration rate using the Chronic Kidney Disease - Epidemiology Collaboration (CKD-EPI) equation.^21^

### Anthropometric evaluation

Anthropometric parameters were evaluated through the measurement of body weight (kg) and height (meters) using electronic scales with a stadiometer (Filizola SA, São Paulo, SP, Brasil), and body mass index (BMI) was calculated and expressed as kg/m^2^.

### Blood pressure and cardiovascular risk assessment

Measurements of systolic BP (SBP) and diastolic BP (DBP) were obtained using a calibrated electronic device (model HEM-705CP, OMRON Healthcare Inc., Illinois). After three readings with a one-minute interval, the mean was calculated and considered for study analysis. The estimation of vascular age was based on the Framingham Heart Study.^22^

### Microvascular reactivity

Microvascular reactivity was evaluated using a Laser Speckle Contrast Image (Pericam PSI System, Perimed, Sweden) along with post-occlusive reactive hyperemia (PORH) for continuous reduction of microvascular endothelium-dependent cutaneous perfusion changes expressed in arbitrary perfusion units (APU). A sphygmomanometer was used on the brachial artery to apply a pressure of 50 mmHg above the SBP for three minutes. After rapid decompression, flow changes were recorded to evaluate the PORH. Increased perfusion (%): (peak - Baseline perfusion) /Baseline perfusion x 100. The area under the curve (AUC) augmentation (%): (PORH-AUC - Baseline AUC) /Baseline AUC x 100.

### Central hemodynamic parameters

The assessment of arterial wave reflection was performed non-invasively using a commercially available tonometry device (SphygmoCor, AtCor Medical, Sydney, Australia). The SphygmoCor system uses a validated generalized transfer function to generate the corresponding central aortic pressures after acquiring 10 sequential waveforms. Aortic systolic pressure (ASP), aortic pulse pressure (APP), augmentation pressure (AP), augmentation index (AIx), SEVR and ejection duration (ED) derived from pulse waveform analysis. SEVR is an index of subendocardial viability that has already been compared with invasive methods and has been considered a measure of myocardial perfusion relative to cardiac overload. In pulse wave analysis, SEVR was defined as SEVR = aortic diastolic area/ aortic systolic area.^6 , 23^

### Statistical analysis

To determine the sample size for this study, we considered the equivalence of variation in flow-mediated dilation (FMD) observed in Bakker’s study (2015).^24^Thus, for a difference of 1.4% in the FMD, standard deviation (SD) of 1.9, study power of 80% and significance of 5%, a minimum number of 30 participants would be necessary. Results were expressed as mean ± SD for continuous variables with normal distribution or median (interquartile range) for non-Gaussian continuous variables. The Shapiro-Wilk test was used to assess normal distribution. The paired t-test compared the control and intervention groups for normal distribution variables, and the Wilcoxon test was performed for variables with non-normal distribution. Categorical variables were presented as frequency and percentage. Pearson coefficient was obtained in correlation tests between continuous variables. The level of significance adopted in the statistical analysis was 5%. Statistical analysis was performed using the Statistical Package for the Social Sciences (SPSS)® version 20 for Windows (SPSS, Chicago, IL).

## Results

For this study, 37 patients with a mean age of 59±7 years were included in the study. After the anthropometric and clinical assessment, it was observed that the majority were overweight with a BMI of 29±4 kg/m2, female (62%), with intermediate cardiovascular risk (14%), SBP>140 mmHg, DBP>80 mmHg, and high total cholesterol ( Table 1 ). The most used classes of antihypertensive drugs were inhibitors of the renin-angiotensin system (48%), thiazide diuretic (36%) and calcium channel blockers (CCB; 16%).

**Table 1:** Baseline characteristics of study subjects

	Total sample (n= 37)
Age (years)	59 ± 7
Cardiovascular risk (%)	14 (10 - 22)
Vascular Age (years)	76 (67 - 86)
Gender ♂ (%)	38
♀ (%)	62
Body Mass Index (Kg/m^2^)	29 ± 4
Waist Circumference (cm) ♂	98 ± 8
♀	92 ± 11
Systolic BP (mmHg)	142 ± 10
Dyastolic BP (mmHg)	83 ± 9
Pulse Pressure (mmHg)	59 ± 11
Mean Arterial Pressure (mmHg)	103 ± 9
**Biochemical Variables**	
Total cholesterol (mg/dl)	203 ± 38
HDL-cholesterol (mg/dl)	56 ± 20
LDL-cholesterol (mg/dl)	121 ± 30
Triglycerides (mg/dl)	110 (78 - 178)
Glucose (mg/dl)	90 ± 8
CKD-EPI (ml/min/1.73m^2^)	80 (67 - 98)
**Antihypertensive treatment, n (%)**	
Angiotensin receptor blockers	29 (78)
Angiotensin-converting enzyme inhibitor	7 (19)
Thiazide diuretic	27 (73)
Calcium channel blocker	12 (32)
Monotherapy	8 (22)
2 drugs	20 (54)
3 drugs	9 (24)

*Results expressed as mean (± Standard Deviation), median (interquartile range) or proportion in categorical variables. BP: blood pressure; HDL: high-density lipoprotein; LDL: low-density lipoprotein; CKD-EPI: Chronic Kidney Disease - Epidemiology Collaboration.*

The BRJ presented high levels of NO_3_‾ and NO_2_‾ in its composition. The serum analysis of NO_3_‾ and NO_2_‾ contents in the control and beetroot phases before and after each intervention is described in Table 2 . No significant differences were observed in NO_3_‾ and NO_2_‾ serum concentrations before the intake of BRJ and water. However, there was a significant increase in serum NO_3_‾ and NO_2_‾ after intervention with BRJ. This increase was approximately three times the baseline value of this phase.

**Table 2:** Serum nitrate and nitrite – Control and beetroot phases

	Control		p-value	Beetroot		p-value
	
	Baseline (n=24)	After (n=24)		Baseline (n=28)	After (n=28)	
Nitrate (µmol/L)	56.4 ± 26.5	65.3 ± 34.9	0.063	59.7 ± 13.6	169.4 ± 76.9	< 0.001
Nitrite (µmol/L)	0.100 ± 0.012	0.095 ± 0.018	0.162	0.099 ± 0.014	0.336 ± 0.159	< 0.001

*Values are presented as the mean ± standard deviation. P value corresponding to the paired t-test in the comparison between baseline and post-intervention values.*

A significant increase in peripheral SBP was observed in the control phase but not in the beetroot phase. There was also an increase in ASP in the control phase and, to a lesser degree, in the beetroot phase. The beetroot phase showed a significant reduction in ED and an increase in SEVR ( Table 3 ).

**Table 3:** Measurements of peripheral blood pressure and central hemodynamic parameters – Control and beetroot phase

	Control		p-value	Beetroot		p-value
	
	Baseline (n=37)	After (n=37)		Baseline (n=37)	After (n=37)	
**Peripheral Blood Pressure**						
Systolic BP (mmHg)	139 ± 9	144 ± 15	0.044	138 ± 13	139 ± 17	0.621
Diastolic BP (mmHg)	83 ± 9	84 ± 9	0.268	84 ± 11	85 ± 11	0.492
Pressure Pulse (mmHg)	56 ± 10	60 ± 13	0.039	54 ± 10	55 11	0.905
MAP (mmHg)	102 ± 8	104 ± 9	0.093	102 ± 11	103 ± 12	0.532
**Central Hemodynamic Parameters by Applanation Tonometry**						
ASP (mmHg)	137 ± 15	143 ± 14	0.003	132 ± 15	136 ± 16	0.061
APP (mmHg)	52 ± 12	56 ± 13	0.007	56 ± 26	57 ± 24	0.736
AP (mmHg)	19 ± 7	21 ± 9	0.009	17 ± 9	19 ± 8	0.278
Augmentation Index (%)	36 (32 - 40)	38 (32 - 43)	0.070	35 (28 - 39)	37 (31 - 41)	0.082
AIx@75 (%)	30 (27 - 34)	32 (26 - 37)	0.442	29 (24 - 34)	31 (24 - 34)	0.751
Ejection duration (%)	35 ± 4	34 ± 4	0.019	37 ± 4	34 4	<0.001
SEVR (%)	155 ± 28	160 ± 28	0.080	149 ± 25	165 ± 30	<0.001

*Data expressed as mean ± SD or median (IQR) when appropriate. P value in the comparison between baseline and post-intervention values corresponding to the paired t-test for variables with normal distribution and the Wilcoxon test for variables with non-normal distribution. BP: blood pressure; MAP: mean arterial pressure; ASP: aortic systolic pressure; APP: aortic pulse pressure; AP: augmentation pressure; Alx@75: Augmentation Index corrected for a heart rate of 75 beats per minute; SEVR: subendocardial viability ratio.*

In the PORH test, the beetroot phase demonstrated a significant increase in % perfusion. The percentage of increase in the AUC of skin perfusion induced by PORH in the control phase decreased after water (control drink) and raised after BRJ intake but not reaching statistical significance ( Table 4 ).

**Table 4:** Measurements of endothelial function evaluation by microvascular reactivity – Control and beetroot phase

	Control		p-value	Beetroot		p-value
	
	Baseline (n=37)	After (n=37)		Baseline (n=37)	After (n=37)	
Basaline perfusion (APU)	30 ± 12	27 ± 10	0.097	33 ± 11	29 ± 9	0.005
Pick (APU)	83 ± 22	73 ± 23	0.001	85 ± 24	76 ± 21	0.005
Increased perfusion (%)	177 (132 - 243)	148 (102 - 212)	0.722	155 (125 - 190)	159 (121 - 227)	0.042
AUC increase (%)	65 ± 34	63 ± 36	0.816	67 ± 28	73 ± 25	0.182

*Data expressed as mean ± SD or median (IQR) when appropriate. P value in the comparison between baseline and post-intervention values corresponding to the paired t-test for variables with normal distribution and the Wilcoxon test for variables with non-normal distribution. APU: arbitrary perfusion unit; AUC: area under the curve.*

In the beetroot phase, the change in SEVR positively correlated with a change in AUC-PORH ( Figure 2 ). The ED in the beetroot phase observed inverse correlations with endothelial function parameters, post-intervention peak (A) and AUC-PORH (B) ( Figure 3 ).

**Figure 2:** Correlation in the beet group between change in the subendocardial viability ratio (∆ SEVR) and change in the area under the curve of post-occlusive reactive hyperemia (∆ AUC-PORH).

**Figure 3:** Correlation in the change beet group (∆) in ejection duration with the post-intervention peak (A) and with the post-intervention area under the post-occlusive reactive hyperemia curve(AUC-PORH) (B).

## Discussion

The present study was conducted to determine the acute effects of dietary NO_3_‾ intake through BRJ, rich in inorganic NO_3_‾, on BP and endothelial function in treated hypertensive patients. Among the main results, attenuation in the peripheral and central BP levels, reduction of ED, increase in SEVR and improvement of vascular function associated with elevated serum NO_3_‾ and NO_2_‾ were observed after a single intake of NO_3_‾ inorganic.

In this study, the BRJ used in the intervention phase presented high levels of NO_3_‾ and NO_2_‾ in its composition. We used fresh vegetables to prepare the BRJ instead of purchasing a more expensive commercial juice. Several studies have used industrialized BRJ with similar NO_3_‾ concentrations but lower NO_2_‾ content than those observed in the current study.^17 , 25^Additionally, the NO_3_‾ concentration was nearly 1.5 times higher than that in the non-industrialized juice used in another recent study.^26^

The serum NO_3_‾ and NO_2_‾ were significantly increased after 150 minutes of intervention with BRJ in this study. Webb et al.^17^evaluated the single intake of 500 ml BRJ and found a rapid increase (16-fold) in circulating NO_3_‾ concentration after the first 30 minutes, with a peak of 1.5 hours and remaining at this level until 6 hours after ingestion. The proportion of circulating NO_3_‾ raise was similar compared to the NO_3_‾ content of this study.

There was a significant increase in peripheral BP in the control phase, which was attenuated in the intervention phase after the ingestion of BRJ. Most studies in normotensive individuals have evaluated chronic consumption of BRJ and have a decrease in BP levels.^27 , 28^Few studies have assessed the effects of BRJ consumption by hypertensive individuals.^15 , 29^Kapil et al.^30^evaluated the intake of BRJ for four weeks in hypertensive patients with and without antihypertensive treatment and found a reduction in SBP and DBP values compared to baseline. Kerley et al.^31^reported significant reductions in BP levels assessed by 24-hour ambulatory BP monitoring (ABPM) after chronic intake of BRJ in treated hypertensive individuals. However, Bondonno et al. (2015) did not observe changes in office BP and ABPM when evaluating hypertensive individuals treated with BRJ intake for one week.^29^

These controversial results in hypertensive individuals could be attributed to the great heterogeneity of the study design concerning the BRJ volume (140mL to 250mL), NO_3_‾ content (6.8 to 12.9 mmol), supplementation time and antihypertensive treatment. Some pharmacological agents, such as renin-angiotensin system inhibitors and CCB, may cause vasodilation by influencing NO synthesis,^32^which could have attenuated the effects of BRJ on BP levels in treated patients in this study. Another factor to be considered is presented in a recent meta-analysis, in which the authors demonstrated that the BP–lowering effects of beetroot might be affected by chronic diseases. A greater reduction in SBP and DBP was observed after beetroot supplementation in unhealthier than healthy participants. Additionally, overweight and obese subjects had a higher response similar to BRJ supplementation.^33^

The behavior of central BP was similar to the peripheral BP, with a significant increase in the control phase, which was not observed in the beetroot phase. To date, there are no studies evaluating central BP after inorganic NO_3_‾ ingestion in individuals with hypertension. Indeed, a few studies that evaluated the central BP after acute ingestion of BRJ were conducted in normotensive individuals, showing a significant reduction in aortic BP.^34 , 35^

In this study, ED showed a significant reduction after ingestion of BRJ. Hughes et al.^34^evaluated the acute effects of BRJ intake by normotensive women and found a gradual reduction of ED after two hours of ingestion. The reduction in ED is related to a less rigid aorta and a decrease in cardiac afterload, and intake of BRJ appeared to improve vascular compliance, which might facilitate cardiac performance.^36^

SEVR is a sensitive marker of subendocardial oxygen supply and demand that correlates with myocardial ischemia.^37^The lower the viability ratio, the lower the cardiac perfusion, which may be related to arterial stiffness. Inflammatory mediators actively participate in vascular injury mechanisms and are increased in all stages of hypertension, and this association accelerates the vascular aging process.^38^In this study, SEVR significantly increased after ingestion of BRJ, and no change was observed in the control phase. To our knowledge, no studies address SEVR in hypertensive individuals submitted to BRJ intake. In agreement with this result, Hughes et al.^34^assessed the acute effects of BRJ intake by young and postmenopausal normotensive women and observed a significant increase in SEVR after 150 and 180 minutes of drinking.

The microvascular reactivity measured by the percentage of perfusion increase in PORH showed a significant increase after ingestion of BRJ, demonstrating an improvement in endothelial function. Thus far, no clinical protocols are evaluating the effects of dietary NO_3_‾ on microvascular reactivity using this methodology in hypertensive individuals. In a study conducted on overweight individuals, three weeks of 70ml BRJ supplementation resulted in no significant difference in peak and PORH index.^39^

Some limitations of this study should be considered. Exclusion criteria made it difficult to enroll participants, especially regarding the use of beta-blockers and statins, drugs widely used in hypertensive patients due to the other comorbidities. However, the study population was more numerous than the estimated sample size and similar to the other studies. The intervention period of 150 minutes in the fasting period may have influenced the BP increase, which was more evident in the control phase. On the other hand, this effect contributed to better observation of the dietary NO3‾ action on BP levels. Finally, we conducted a single-dose intervention; therefore, our findings cannot be compared with medium and long-term effects.

## Conclusion

The intake of BRJ resulted in acute benefits on vascular parameters in hypertensive individuals, leading to greater subendocardial viability, higher performance in myocardial contraction and improvement in endothelial function. This was the first study that applied different methods to evaluate vascular parameters and demonstrated the beneficial effects of the single intake of BRJ in treated hypertensive adults. Nevertheless, further studies are needed to assess the efficacy of the NO_3_-NO_2_-NO pathway, especially in subjects with hypertension and other risk factors for cardiovascular disease.
